# AGELESS HORIZONS: IMPACT OF AI, CLIMATE CHANGE, AND EPIGENETICS ON GERONTOLOGICAL SYNDROMES

**DOI:** 10.1093/geroni/igae098.0435

**Published:** 2024-12-31

**Authors:** Todd Manini, Christopher Kaufmann

**Affiliations:** Chair; Co-Chair

## Abstract

This interdisciplinary symposium will provide the audience an eclectic, contemporary view of gerontology syndromes. It brings together researchers in epidemiology, built environment to natural hazards, new epigenetic age measures, climate events, artificial intelligence (AI) and gerontological syndromes. In this symposium, the audience will learn about how the literature is using AI to study the 5 M’s of Geriatrics — Mind, Mobility, Medications, what Matters most and Multicomplexity. This topic is complemented by using new AI methods with Latent Dirichlet Allocation to analyze clinical notes to better estimate the frailty syndrome. Results from older adults undergoing Transcatheter Aortic Valve Replacement suggest a substantial improvement in identifying frail older adults compared to an electronic record approach only. Next, new hallmark measures of biological aging using epigenetic age are studied as a potential mechanism by which biopsychosocial factors could influencing pain and mobility in older adults. Lastly, there are significant adverse health outcomes due to environmental shocks of extreme weather and climate events. Older adults are known to have a greater health susceptibility to these events. However, the impact on health can happen in even deeper ways by modifying mobility after the acute effects have ceased. Mobility resilience signifies a capacity to adapt and maintain community interactions following environmental shocks. Results from hurricane Ian in 2022 suggest that neighborhoods with a higher density of older adults are less mobility resilient. Today’s advances in AI and access to unique data sources are on the horizon and will open new approaches to studying gerontological syndromes.

